# Bromidotetra­kis­(2-ethyl-1*H*-imidazole-κ*N* ^3^)copper(II) bromide

**DOI:** 10.1107/S1600536812047447

**Published:** 2012-11-24

**Authors:** Sylwia Godlewska, Harald Kelm, Hans-Jörg Krüger, Anna Dołęga

**Affiliations:** a Department of Inorganic Chemistry, Faculty of Chemistry, Gdansk University of Technology, 11/12 G. Narutowicz Street, 80233-PL Gdańsk, Poland; b Fachbereich Chemie, Technische Universität Kaiserslautern, Erwin-Schrödinger Strasse 54, 67663 Kaiserslautern, Germany

## Abstract

The Cu^II^ ion in the title mol­ecular salt, [CuBr(C_5_H_8_N_2_)_4_]Br, is coordinated in a square-pyramidal geometry by four N atoms of imidazole ligands and one bromide anion in the apical position. In the crystal, the ions are linked by N—H⋯Br hydrogen bonds involving both the coordinating and the free bromide species as acceptors. A C—H⋯Br inter­action is also observed. Overall, a three-dimensional network results.

## Related literature

For more copper(II) complexes with bromido and imidazole ligands, see: Godlewska *et al.* (2011 ▶); Hossaini Sadr *et al.* (2004 ▶); Li *et al.* (2007 ▶); Liu *et al.* (2007 ▶); Näther *et al.* (2002*a* ▶,*b* ▶); Parker & Breneman (1995 ▶).
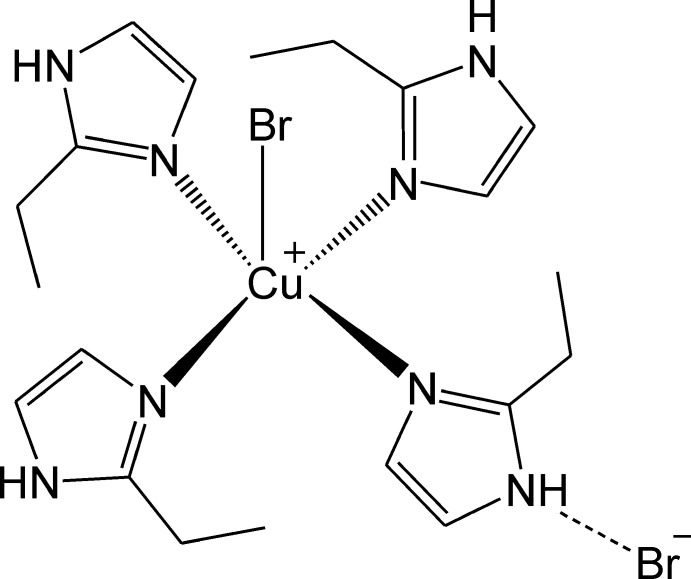


## Experimental

### 

#### Crystal data

[CuBr(C_5_H_8_N_2_)_4_]Br
*M* *_r_* = 607.90
Monoclinic, 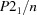

*a* = 10.1771 (2) Å
*b* = 19.9230 (3) Å
*c* = 12.5723 (2) Å
β = 90.386 (2)°
*V* = 2549.08 (8) Å^3^
*Z* = 4
Cu *K*α radiation
μ = 5.06 mm^−1^
*T* = 150 K
0.21 × 0.20 × 0.05 mm

#### Data collection

Oxford Diffraction Xcalibur (Sapphire3, Gemini ultra) diffractometer
Absorption correction: multi-scan (*CrysAlis PRO*; Oxford Diffraction, 2010 ▶) *T*_min_ = 0.416, *T*_max_ = 0.786
9229 measured reflections
4058 independent reflections
3719 reflections with *I* > 2σ(*I*)
*R*_int_ = 0.020

#### Refinement

*R*[*F* ^2^ > 2σ(*F* ^2^)] = 0.024
*wR*(*F* ^2^) = 0.060
*S* = 1.05
4058 reflections
284 parameters
H-atom parameters constrained
Δρ_max_ = 0.42 e Å^−3^
Δρ_min_ = −0.34 e Å^−3^

### 

Data collection: *CrysAlis PRO* (Oxford Diffraction, 2010 ▶); cell refinement: *CrysAlis PRO*; data reduction: *CrysAlis PRO*; program(s) used to solve structure: *SHELXS97* (Sheldrick, 2008 ▶); program(s) used to refine structure: *SHELXL97* (Sheldrick, 2008 ▶); molecular graphics: *SHELXTL* (Sheldrick, 2008 ▶); software used to prepare material for publication: *SHELXL97*.

## Supplementary Material

Crystal structure: contains datablock(s) I, global. DOI: 10.1107/S1600536812047447/hb6993sup1.cif

Structure factors: contains datablock(s) I. DOI: 10.1107/S1600536812047447/hb6993Isup2.hkl

Additional supplementary materials:  crystallographic information; 3D view; checkCIF report

## Figures and Tables

**Table 1:** Selected bond lengths (Å)

Cu1—N3	1.9914 (19)
Cu1—N7	1.9918 (19)
Cu1—N5	2.014 (2)
Cu1—N1	2.0250 (19)
Cu1—Br1	3.0125 (4)

**Table 2:** Hydrogen-bond geometry (Å, °)

*D*—H⋯*A*	*D*—H	H⋯*A*	*D*⋯*A*	*D*—H⋯*A*
N2—H2⋯Br1^i^	0.88	2.56	3.405 (2)	161
N4—H4⋯Br2^ii^	0.88	2.46	3.336 (2)	176
N6—H6*A*⋯Br2	0.88	2.47	3.302 (2)	157
N8—H8⋯Br1^iii^	0.88	2.56	3.432 (2)	172
C17—H17⋯Br2^iv^	0.95	2.89	3.653 (3)	138

Symmetry codes: (i) 
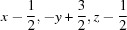
; (ii) 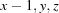; (iii) 
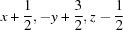
; (iv) 
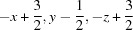
.

**Fig. 1.**
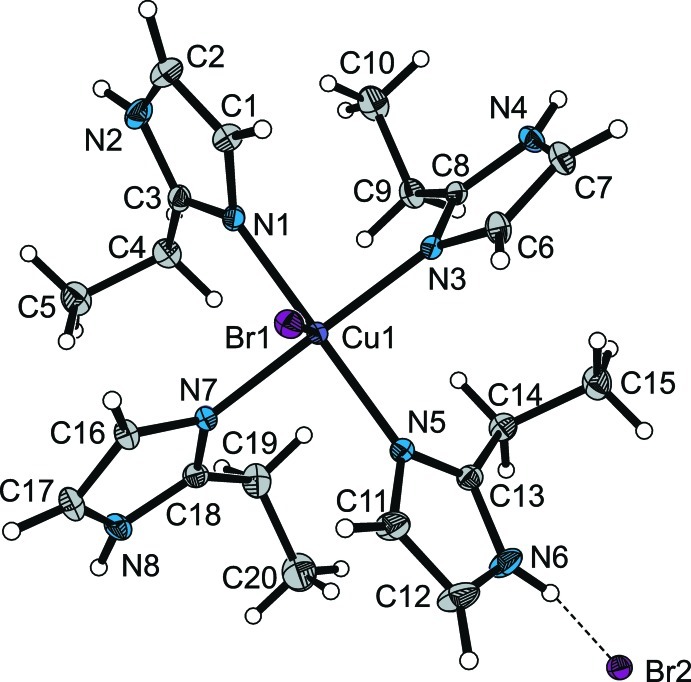
The molecular structure of (I) with displacement ellipsoids drawn at the 30% probability level. Hydrogen bond indicated by the dashed line.

**Fig. 2.**
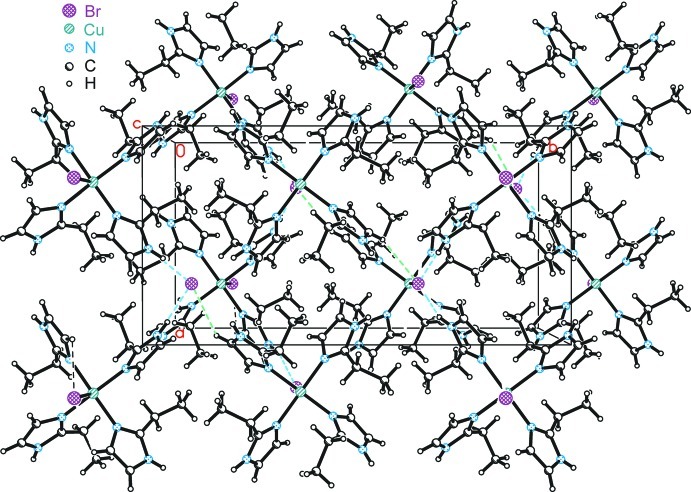
The crystal packing of (I). NH—Br hydrogen bonds indicated as blue dashed lines and CH—Br contacts as green dashed lines.

## supplementary crystallographic information

### Comment

The title compound, (I), is the third in a series of similar complex compounds (Godlewska *et al.* (2011)). The complex cation features square pyramidal geometry around Cu atom with four N atoms in the basal plane and apical bromide ligand. The deviation of N—Cu—N angles from the values of 90 and 180 degrees are very small and such is the deviation of Cu atom (0.1077 Å) from the basal plane formed by the four nitrogen atoms of imidazole ligands. The complex cations and Br2 anions form a three-dimensional network of intervowen NH···Br and CH···Br interactions that results in a "compact" packing of the interacting species and the relatively large density of the obtained crystals.

The asymmetric unit of (I) is shown in Fig. 1 and packing diagram is presented in Fig.2.

### Experimental

The title compound was prepared by adding a solution of 0.223 g (1 mmol) copper(II) bromide in 4 ml of methanol to a solution of 0.433 g (4.5 mmol) 2-ethylimidazole in 2 ml of methanol. After few days violet crystals were obtained by slow evaporation of solvent from the reaction mixture.

### Refinement

All C–H hydrogen atoms were refined as riding on carbon atoms with methyl C–H = 0.99 Å, methylene C–H = 0.98 Å, aromatic C–H = 0.95 Å and *U*_iso_(H)=1.5*U*_eq_(C) for methyl groups and 1.2 *U*_eq_(C) for the rest of H atoms.

### Crystal data

**Table:**

[CuBr(C_5_H_8_N_2_)_4_]Br	*F*(000) = 1228
*M**_r_* = 607.90	*D*_x_ = 1.584 Mg m^−^^3^
Monoclinic, *P*2_1_/*n*	Cu *K*α radiation, λ = 1.54184 Å
Hall symbol: -P 2yn	Cell parameters from 6482 reflections
*a* = 10.1771 (2) Å	θ = 3.5–62.6°
*b* = 19.9230 (3) Å	µ = 5.06 mm^−^^1^
*c* = 12.5723 (2) Å	*T* = 150 K
β = 90.386 (2)°	Indifferent fragment, violet
*V* = 2549.08 (8) Å^3^	0.21 × 0.20 × 0.05 mm
*Z* = 4	

### Data collection

**Table:**

Oxford Diffraction Xcalibur (Sapphire3, Gemini ultra) diffractometer	4058 independent reflections
Radiation source: fine-focus sealed tube	3719 reflections with *I* > 2σ(*I*)
Mirror monochromator	*R*_int_ = 0.020
Detector resolution: 16.1399 pixels mm^-1^	θ_max_ = 62.7°, θ_min_ = 4.2°
ω scans	*h* = −11→10
Absorption correction: multi-scan (*CrysAlis PRO*; Oxford Diffraction, 2010)	*k* = −22→18
*T*_min_ = 0.416, *T*_max_ = 0.786	*l* = −14→10
9229 measured reflections	

### Refinement

**Table:**

Refinement on *F*^2^	Primary atom site location: structure-invariant direct methods
Least-squares matrix: full	Secondary atom site location: difference Fourier map
*R*[*F*^2^ > 2σ(*F*^2^)] = 0.024	Hydrogen site location: inferred from neighbouring sites
*wR*(*F*^2^) = 0.060	H-atom parameters constrained
*S* = 1.05	*w* = 1/[σ^2^(*F*_o_^2^) + (0.0323*P*)^2^ + 1.0444*P*] where *P* = (*F*_o_^2^ + 2*F*_c_^2^)/3
4058 reflections	(Δ/σ)_max_ = 0.002
284 parameters	Δρ_max_ = 0.42 e Å^−^^3^
0 restraints	Δρ_min_ = −0.34 e Å^−^^3^

### Special details

**Table:**

Geometry. All s.u.'s (except the s.u. in the dihedral angle between two l.s. planes) are estimated using the full covariance matrix. The cell s.u.'s are taken into account individually in the estimation of s.u.'s in distances, angles and torsion angles; correlations between s.u.'s in cell parameters are only used when they are defined by crystal symmetry. An approximate (isotropic) treatment of cell s.u.'s is used for estimating s.u.'s involving l.s. planes.
Refinement. Refinement of *F*^2^ against ALL reflections. The weighted *R*-factor *wR* and goodness of fit *S* are based on *F*^2^, conventional *R*-factors *R* are based on *F*, with *F* set to zero for negative *F*^2^. The threshold expression of *F*^2^ > 2σ(*F*^2^) is used only for calculating *R*-factors(gt) *etc*. and is not relevant to the choice of reflections for refinement. *R*-factors based on *F*^2^ are statistically about twice as large as those based on *F*, and *R*- factors based on ALL data will be even larger.

### Fractional atomic coordinates and isotropic or equivalent isotropic displacement parameters (Å^2^)

**Table:**

	*x*	*y*	*z*	*U*_iso_*/*U*_eq_	
Cu1	0.24721 (3)	0.861967 (16)	0.79905 (3)	0.01634 (9)	
Br1	0.23840 (2)	0.845061 (13)	1.036999 (19)	0.02191 (8)	
N1	0.08773 (19)	0.80290 (10)	0.77538 (16)	0.0205 (4)	
N2	−0.0633 (2)	0.73282 (11)	0.71786 (18)	0.0298 (5)	
H2	−0.1047	0.7035	0.6774	0.036*	
C1	−0.0142 (2)	0.80070 (13)	0.8483 (2)	0.0257 (6)	
H1	−0.0181	0.8257	0.9126	0.031*	
C2	−0.1070 (3)	0.75730 (14)	0.8132 (2)	0.0321 (6)	
H2A	−0.1867	0.7460	0.8478	0.038*	
C3	0.0542 (2)	0.76125 (12)	0.6966 (2)	0.0235 (5)	
C4	0.1271 (3)	0.74770 (14)	0.5965 (2)	0.0284 (6)	
H4A	0.1946	0.7829	0.5871	0.034*	
H4B	0.0651	0.7507	0.5357	0.034*	
C5	0.1940 (3)	0.67900 (15)	0.5945 (2)	0.0376 (7)	
H5A	0.2570	0.6759	0.6536	0.056*	
H5B	0.2403	0.6734	0.5270	0.056*	
H5C	0.1276	0.6437	0.6016	0.056*	
N3	0.12993 (18)	0.94198 (10)	0.79926 (15)	0.0185 (4)	
N4	−0.0231 (2)	1.01429 (10)	0.75619 (17)	0.0234 (5)	
H4	−0.0838	1.0353	0.7188	0.028*	
C6	0.1095 (2)	0.98466 (12)	0.8846 (2)	0.0244 (5)	
H6	0.1547	0.9829	0.9509	0.029*	
C7	0.0141 (3)	1.02935 (13)	0.8579 (2)	0.0271 (6)	
H7	−0.0200	1.0642	0.9013	0.033*	
C8	0.0490 (2)	0.96163 (12)	0.72257 (19)	0.0192 (5)	
C9	0.0401 (3)	0.93264 (13)	0.6135 (2)	0.0270 (6)	
H9A	0.1069	0.8968	0.6062	0.032*	
H9B	0.0604	0.9681	0.5609	0.032*	
C10	−0.0951 (3)	0.90355 (15)	0.5886 (3)	0.0417 (8)	
H10A	−0.1172	0.8695	0.6418	0.063*	
H10B	−0.0943	0.8829	0.5178	0.063*	
H10C	−0.1608	0.9395	0.5900	0.063*	
N5	0.40712 (19)	0.92118 (10)	0.81018 (16)	0.0208 (4)	
N6	0.5637 (2)	0.99454 (12)	0.78382 (19)	0.0340 (6)	
H6A	0.6086	1.0277	0.7556	0.041*	
C11	0.5028 (3)	0.91305 (14)	0.8881 (2)	0.0323 (6)	
H11	0.5007	0.8808	0.9437	0.039*	
C12	0.5992 (3)	0.95818 (17)	0.8722 (2)	0.0414 (8)	
H12	0.6766	0.9638	0.9140	0.050*	
C13	0.4484 (2)	0.97063 (12)	0.7479 (2)	0.0227 (5)	
C14	0.3819 (3)	0.99879 (13)	0.6518 (2)	0.0268 (6)	
H14A	0.3141	0.9667	0.6268	0.032*	
H14B	0.4473	1.0039	0.5944	0.032*	
C15	0.3173 (3)	1.06667 (14)	0.6727 (2)	0.0355 (7)	
H15A	0.2571	1.0627	0.7329	0.053*	
H15B	0.2681	1.0808	0.6093	0.053*	
H15C	0.3851	1.1000	0.6893	0.053*	
N7	0.36498 (18)	0.78339 (10)	0.77723 (16)	0.0189 (4)	
N8	0.5199 (2)	0.71775 (11)	0.71834 (18)	0.0270 (5)	
H8	0.5820	0.7013	0.6776	0.032*	
C16	0.3837 (2)	0.73151 (12)	0.8490 (2)	0.0245 (5)	
H16	0.3364	0.7253	0.9132	0.029*	
C17	0.4800 (3)	0.69128 (13)	0.8130 (2)	0.0302 (6)	
H17	0.5136	0.6522	0.8467	0.036*	
C18	0.4486 (2)	0.77323 (13)	0.6977 (2)	0.0237 (5)	
C19	0.4629 (3)	0.81532 (15)	0.6011 (2)	0.0340 (6)	
H19A	0.3952	0.8510	0.6017	0.041*	
H19B	0.4476	0.7872	0.5373	0.041*	
C20	0.5988 (3)	0.84754 (18)	0.5942 (3)	0.0557 (10)	
H20A	0.6180	0.8718	0.6603	0.084*	
H20B	0.6005	0.8790	0.5342	0.084*	
H20C	0.6651	0.8126	0.5835	0.084*	
Br2	0.73566 (2)	1.087768 (13)	0.61807 (2)	0.02419 (8)	

### Atomic displacement parameters (Å^2^)

**Table:**

	*U*^11^	*U*^22^	*U*^33^	*U*^12^	*U*^13^	*U*^23^
Cu1	0.01598 (17)	0.01449 (17)	0.01854 (18)	0.00071 (13)	−0.00093 (13)	0.00029 (13)
Br1	0.02224 (14)	0.02382 (14)	0.01968 (14)	0.00267 (10)	0.00118 (10)	0.00369 (10)
N1	0.0216 (10)	0.0164 (10)	0.0233 (11)	−0.0011 (8)	−0.0030 (8)	0.0007 (8)
N2	0.0298 (12)	0.0262 (12)	0.0333 (13)	−0.0099 (10)	−0.0089 (10)	−0.0016 (10)
C1	0.0244 (13)	0.0288 (14)	0.0240 (13)	−0.0035 (11)	0.0002 (10)	0.0001 (11)
C2	0.0276 (14)	0.0350 (15)	0.0336 (15)	−0.0101 (12)	−0.0004 (12)	0.0033 (13)
C3	0.0247 (13)	0.0185 (12)	0.0272 (13)	−0.0005 (10)	−0.0082 (10)	0.0025 (10)
C4	0.0325 (14)	0.0301 (14)	0.0226 (13)	0.0025 (12)	−0.0063 (11)	−0.0050 (11)
C5	0.0433 (17)	0.0336 (16)	0.0359 (16)	0.0049 (13)	−0.0065 (13)	−0.0115 (13)
N3	0.0187 (10)	0.0160 (10)	0.0208 (10)	−0.0006 (8)	−0.0001 (8)	0.0006 (8)
N4	0.0228 (10)	0.0220 (11)	0.0253 (11)	0.0072 (9)	−0.0009 (9)	0.0045 (9)
C6	0.0334 (14)	0.0208 (13)	0.0190 (13)	0.0033 (11)	0.0009 (10)	−0.0029 (10)
C7	0.0346 (14)	0.0221 (13)	0.0247 (13)	0.0080 (11)	0.0042 (11)	−0.0015 (11)
C8	0.0187 (12)	0.0172 (12)	0.0218 (12)	−0.0002 (10)	−0.0006 (10)	0.0026 (10)
C9	0.0326 (14)	0.0262 (14)	0.0222 (13)	0.0048 (11)	−0.0035 (11)	0.0026 (11)
C10	0.0432 (17)	0.0373 (17)	0.0443 (18)	0.0062 (14)	−0.0207 (14)	−0.0104 (14)
N5	0.0184 (10)	0.0196 (10)	0.0243 (11)	−0.0007 (8)	0.0001 (8)	0.0020 (9)
N6	0.0286 (12)	0.0348 (13)	0.0384 (14)	−0.0148 (10)	−0.0038 (10)	0.0108 (11)
C11	0.0269 (14)	0.0379 (16)	0.0320 (15)	−0.0047 (12)	−0.0083 (12)	0.0128 (13)
C12	0.0294 (15)	0.055 (2)	0.0393 (17)	−0.0153 (14)	−0.0138 (13)	0.0157 (15)
C13	0.0212 (12)	0.0240 (13)	0.0230 (13)	−0.0013 (10)	0.0024 (10)	−0.0025 (10)
C14	0.0288 (14)	0.0273 (14)	0.0243 (14)	−0.0023 (11)	0.0034 (11)	0.0047 (11)
C15	0.0418 (16)	0.0286 (15)	0.0362 (16)	0.0019 (13)	0.0009 (13)	0.0108 (13)
N7	0.0191 (10)	0.0164 (10)	0.0213 (10)	0.0017 (8)	0.0003 (8)	0.0000 (8)
N8	0.0240 (11)	0.0256 (12)	0.0314 (12)	0.0075 (9)	0.0041 (9)	−0.0071 (10)
C16	0.0290 (13)	0.0213 (13)	0.0231 (13)	0.0039 (11)	−0.0010 (11)	0.0014 (10)
C17	0.0371 (15)	0.0222 (14)	0.0311 (15)	0.0091 (12)	−0.0040 (12)	0.0003 (11)
C18	0.0215 (12)	0.0237 (13)	0.0259 (13)	0.0007 (10)	0.0013 (10)	−0.0062 (11)
C19	0.0445 (16)	0.0313 (15)	0.0264 (14)	0.0022 (13)	0.0115 (12)	−0.0002 (12)
C20	0.050 (2)	0.047 (2)	0.070 (2)	0.0011 (16)	0.0324 (18)	0.0124 (18)
Br2	0.02263 (14)	0.02361 (14)	0.02629 (15)	−0.00013 (10)	−0.00150 (10)	0.00541 (10)

### Geometric parameters (Å, º)

**Table:**

Cu1—N3	1.9914 (19)	C10—H10B	0.9800
Cu1—N7	1.9918 (19)	C10—H10C	0.9800
Cu1—N5	2.014 (2)	N5—C13	1.328 (3)
Cu1—N1	2.0250 (19)	N5—C11	1.386 (3)
Cu1—Br1	3.0125 (4)	N6—C13	1.341 (3)
N1—C3	1.334 (3)	N6—C12	1.373 (4)
N1—C1	1.390 (3)	N6—H6A	0.8800
N2—C3	1.351 (3)	C11—C12	1.346 (4)
N2—C2	1.371 (4)	C11—H11	0.9500
N2—H2	0.8800	C12—H12	0.9500
C1—C2	1.352 (4)	C13—C14	1.491 (4)
C1—H1	0.9500	C14—C15	1.527 (4)
C2—H2A	0.9500	C14—H14A	0.9900
C3—C4	1.490 (4)	C14—H14B	0.9900
C4—C5	1.529 (4)	C15—H15A	0.9800
C4—H4A	0.9900	C15—H15B	0.9800
C4—H4B	0.9900	C15—H15C	0.9800
C5—H5A	0.9800	N7—C18	1.333 (3)
C5—H5B	0.9800	N7—C16	1.385 (3)
C5—H5C	0.9800	N8—C18	1.347 (3)
N3—C8	1.323 (3)	N8—C17	1.365 (4)
N3—C6	1.386 (3)	N8—H8	0.8800
N4—C8	1.350 (3)	C16—C17	1.348 (4)
N4—C7	1.364 (3)	C16—H16	0.9500
N4—H4	0.8800	C17—H17	0.9500
C6—C7	1.358 (4)	C18—C19	1.483 (4)
C6—H6	0.9500	C19—C20	1.528 (4)
C7—H7	0.9500	C19—H19A	0.9900
C8—C9	1.490 (4)	C19—H19B	0.9900
C9—C10	1.524 (4)	C20—H20A	0.9800
C9—H9A	0.9900	C20—H20B	0.9800
C9—H9B	0.9900	C20—H20C	0.9800
C10—H10A	0.9800		
			
N3—Cu1—N7	172.13 (8)	C9—C10—H10B	109.5
N3—Cu1—N5	90.87 (8)	H10A—C10—H10B	109.5
N7—Cu1—N5	89.04 (8)	C9—C10—H10C	109.5
N3—Cu1—N1	89.17 (8)	H10A—C10—H10C	109.5
N7—Cu1—N1	90.31 (8)	H10B—C10—H10C	109.5
N5—Cu1—N1	175.54 (8)	C13—N5—C11	106.2 (2)
N3—Cu1—Br1	93.80 (6)	C13—N5—Cu1	130.70 (17)
N7—Cu1—Br1	94.06 (6)	C11—N5—Cu1	122.98 (17)
N5—Cu1—Br1	91.48 (6)	C13—N6—C12	108.1 (2)
N1—Cu1—Br1	92.97 (6)	C13—N6—H6A	126.0
C3—N1—C1	106.4 (2)	C12—N6—H6A	126.0
C3—N1—Cu1	132.14 (17)	C12—C11—N5	109.1 (2)
C1—N1—Cu1	121.50 (16)	C12—C11—H11	125.5
C3—N2—C2	108.4 (2)	N5—C11—H11	125.5
C3—N2—H2	125.8	C11—C12—N6	106.5 (2)
C2—N2—H2	125.8	C11—C12—H12	126.8
C2—C1—N1	109.1 (2)	N6—C12—H12	126.8
C2—C1—H1	125.4	N5—C13—N6	110.1 (2)
N1—C1—H1	125.4	N5—C13—C14	127.8 (2)
C1—C2—N2	106.4 (2)	N6—C13—C14	122.0 (2)
C1—C2—H2A	126.8	C13—C14—C15	112.8 (2)
N2—C2—H2A	126.8	C13—C14—H14A	109.0
N1—C3—N2	109.6 (2)	C15—C14—H14A	109.0
N1—C3—C4	127.8 (2)	C13—C14—H14B	109.0
N2—C3—C4	122.5 (2)	C15—C14—H14B	109.0
C3—C4—C5	113.6 (2)	H14A—C14—H14B	107.8
C3—C4—H4A	108.8	C14—C15—H15A	109.5
C5—C4—H4A	108.8	C14—C15—H15B	109.5
C3—C4—H4B	108.8	H15A—C15—H15B	109.5
C5—C4—H4B	108.8	C14—C15—H15C	109.5
H4A—C4—H4B	107.7	H15A—C15—H15C	109.5
C4—C5—H5A	109.5	H15B—C15—H15C	109.5
C4—C5—H5B	109.5	C18—N7—C16	106.9 (2)
H5A—C5—H5B	109.5	C18—N7—Cu1	127.62 (17)
C4—C5—H5C	109.5	C16—N7—Cu1	125.24 (16)
H5A—C5—H5C	109.5	C18—N8—C17	108.8 (2)
H5B—C5—H5C	109.5	C18—N8—H8	125.6
C8—N3—C6	106.7 (2)	C17—N8—H8	125.6
C8—N3—Cu1	127.30 (16)	C17—C16—N7	108.8 (2)
C6—N3—Cu1	125.82 (16)	C17—C16—H16	125.6
C8—N4—C7	108.5 (2)	N7—C16—H16	125.6
C8—N4—H4	125.8	C16—C17—N8	106.5 (2)
C7—N4—H4	125.8	C16—C17—H17	126.7
C7—C6—N3	108.7 (2)	N8—C17—H17	126.7
C7—C6—H6	125.6	N7—C18—N8	109.0 (2)
N3—C6—H6	125.6	N7—C18—C19	126.5 (2)
C6—C7—N4	106.3 (2)	N8—C18—C19	124.5 (2)
C6—C7—H7	126.8	C18—C19—C20	112.2 (3)
N4—C7—H7	126.8	C18—C19—H19A	109.2
N3—C8—N4	109.8 (2)	C20—C19—H19A	109.2
N3—C8—C9	126.1 (2)	C18—C19—H19B	109.2
N4—C8—C9	124.0 (2)	C20—C19—H19B	109.2
C8—C9—C10	112.7 (2)	H19A—C19—H19B	107.9
C8—C9—H9A	109.1	C19—C20—H20A	109.5
C10—C9—H9A	109.1	C19—C20—H20B	109.5
C8—C9—H9B	109.1	H20A—C20—H20B	109.5
C10—C9—H9B	109.1	C19—C20—H20C	109.5
H9A—C9—H9B	107.8	H20A—C20—H20C	109.5
C9—C10—H10A	109.5	H20B—C20—H20C	109.5
			
N3—Cu1—N1—C3	118.9 (2)	N3—Cu1—N5—C13	−57.2 (2)
N7—Cu1—N1—C3	−53.2 (2)	N7—Cu1—N5—C13	115.0 (2)
Br1—Cu1—N1—C3	−147.3 (2)	Br1—Cu1—N5—C13	−151.0 (2)
N3—Cu1—N1—C1	−60.63 (19)	N3—Cu1—N5—C11	126.8 (2)
N7—Cu1—N1—C1	127.22 (19)	N7—Cu1—N5—C11	−61.0 (2)
Br1—Cu1—N1—C1	33.14 (18)	Br1—Cu1—N5—C11	33.0 (2)
C3—N1—C1—C2	0.7 (3)	C13—N5—C11—C12	0.7 (3)
Cu1—N1—C1—C2	−179.70 (18)	Cu1—N5—C11—C12	177.6 (2)
N1—C1—C2—N2	−0.5 (3)	N5—C11—C12—N6	−0.2 (4)
C3—N2—C2—C1	0.1 (3)	C13—N6—C12—C11	−0.5 (4)
C1—N1—C3—N2	−0.6 (3)	C11—N5—C13—N6	−1.0 (3)
Cu1—N1—C3—N2	179.83 (16)	Cu1—N5—C13—N6	−177.53 (18)
C1—N1—C3—C4	177.1 (2)	C11—N5—C13—C14	179.6 (3)
Cu1—N1—C3—C4	−2.5 (4)	Cu1—N5—C13—C14	3.0 (4)
C2—N2—C3—N1	0.3 (3)	C12—N6—C13—N5	1.0 (3)
C2—N2—C3—C4	−177.5 (2)	C12—N6—C13—C14	−179.6 (3)
N1—C3—C4—C5	107.0 (3)	N5—C13—C14—C15	104.3 (3)
N2—C3—C4—C5	−75.6 (3)	N6—C13—C14—C15	−75.1 (3)
N5—Cu1—N3—C8	117.5 (2)	N5—Cu1—N7—C18	−62.3 (2)
N1—Cu1—N3—C8	−58.1 (2)	N1—Cu1—N7—C18	113.3 (2)
Br1—Cu1—N3—C8	−151.00 (19)	Br1—Cu1—N7—C18	−153.7 (2)
N5—Cu1—N3—C6	−68.5 (2)	N5—Cu1—N7—C16	111.1 (2)
N1—Cu1—N3—C6	115.9 (2)	N1—Cu1—N7—C16	−73.3 (2)
Br1—Cu1—N3—C6	23.00 (19)	Br1—Cu1—N7—C16	19.70 (19)
C8—N3—C6—C7	0.8 (3)	C18—N7—C16—C17	1.1 (3)
Cu1—N3—C6—C7	−174.24 (17)	Cu1—N7—C16—C17	−173.44 (17)
N3—C6—C7—N4	−0.4 (3)	N7—C16—C17—N8	−0.7 (3)
C8—N4—C7—C6	−0.2 (3)	C18—N8—C17—C16	0.0 (3)
C6—N3—C8—N4	−0.9 (3)	C16—N7—C18—N8	−1.1 (3)
Cu1—N3—C8—N4	174.05 (15)	Cu1—N7—C18—N8	173.31 (16)
C6—N3—C8—C9	177.0 (2)	C16—N7—C18—C19	178.9 (2)
Cu1—N3—C8—C9	−8.0 (3)	Cu1—N7—C18—C19	−6.7 (4)
C7—N4—C8—N3	0.7 (3)	C17—N8—C18—N7	0.7 (3)
C7—N4—C8—C9	−177.3 (2)	C17—N8—C18—C19	−179.3 (3)
N3—C8—C9—C10	119.5 (3)	N7—C18—C19—C20	116.9 (3)
N4—C8—C9—C10	−62.9 (3)	N8—C18—C19—C20	−63.1 (4)

### Hydrogen-bond geometry (Å, º)

**Table:**

*D*—H···*A*	*D*—H	H···*A*	*D*···*A*	*D*—H···*A*
N2—H2···Br1^i^	0.88	2.56	3.405 (2)	161
N4—H4···Br2^ii^	0.88	2.46	3.336 (2)	176
N6—H6*A*···Br2	0.88	2.47	3.302 (2)	157
N8—H8···Br1^iii^	0.88	2.56	3.432 (2)	172
C17—H17···Br2^iv^	0.95	2.89	3.653 (3)	138

Symmetry codes: (i) *x*−1/2, −*y*+3/2, *z*−1/2; (ii) *x*−1, *y*, *z*; (iii) *x*+1/2, −*y*+3/2, *z*−1/2; (iv) −*x*+3/2, *y*−1/2, −*z*+3/2.
